# Hemolytic Uremic Syndrome Outbreak in Adults and Shiga Toxin–Producing *Escherichia coli* Negative for Locus of Enterocyte Effacement, France, 2025

**DOI:** 10.3201/eid3204.251417

**Published:** 2026-04

**Authors:** Justine de Larminat, Kevin La, Philippe Bidet, André Birgy, Sandrine Liguori, Pierre Phlipaux, Celine Courroux, Florence Crombé, Jorge Blanco, Paul Coppo, Antoine Dossier, Laurence Armand-Lefevre, François Xavier Weill, Carolina Silva Nodari, Gabrielle Jones, Aurelie Cointe, Stéphane Bonacorsi

**Affiliations:** *Escherichia coli* National Reference Center, Robert-Debré University Hospital, Assistance Publique–Hôpitaux de Paris, Paris, France (J. de Larminat, K. La, P. Bidet, A. Birgy, S. Liguori, P. Phlipaux, C. Courroux, A. Cointe, S. Bonacorsi); Infection, Antimicrobien, Modélisation, Évolution, INSERM, Université Paris Cité, Paris (J. de Larminat, K. La, P. Bidet, A. Birgy, L. Armand-Lefevre, A. Cointe, S. Bonacorsi); Universitair Ziekenhuis Brussel, Brussels, Belgium (F. Crombé); Vrije Universiteit Brussel–Campus Jette, Brussels (F. Crombé); University of Santiago de Compostela, Lugo, Spain (J. Blanco); Hôpital Saint-Antoine, Paris (P. Coppo); Sorbonne Université, Paris (P. Coppo); Hôpital Bichat–Claude-Bernard, APHP, Paris (A. Dossier, L. Armand-Lefevre); Université Paris Diderot UFR de Médecine Site Xavier-Bichat, Paris (L. Armand-Lefevre); Institut Pasteur, Université Paris Cité, Paris (F.X. Weill, C. Silva Nodari); Santé Publique France, Saint-Maurice, France (G. Jones)

**Keywords:** *Escherichia coli*, Shiga toxin–producing *Escherichia coli*, STEC, bacteria, hemolytic uremic syndrome, HUS, enteric infections, locus of enterocyte effacement, diarrhea, outbreak, elderly, France

## Abstract

In January 2025, the *Escherichia coli* National Reference Center of France detected an outbreak of hemolytic uremic syndrome (HUS) in adults, caused by Shiga toxin–producing *E. coli* negative for locus of enterocyte effacement (LEE). The outbreak included 18 confirmed cases of *E. coli* infection, 5 probable or possible cases detected by in-house specific PCR, and 2 additional cases from Scotland and Belgium. Whole-genome sequencing identified the outbreak strain as O77 g:K92:H18, belonging to phylogroup D; the strain harbored the Shiga toxin 2 gene variant *stx2d-*073-C165-02 and a 134-kb plasmid with enterotoxin genes (*estb-STb2* and *eltAB*). Epidemiologic investigation implicated raw-milk cheese as the contamination source. The strain represents a singular hybrid pathotype of Shiga toxin–producing and enterotoxigenic *E. coli*, expressing a K92 capsule with known cross-immunogenicity to *Neisseria meningitidis* group C, which could explain the absence of pediatric cases. Related strains have been identified in international databases since 2005, suggesting global emergence.

Hemolytic uremic syndrome (HUS) is the most severe and potentially lethal complication of infections caused by Shiga toxin–producing *E. coli* (STEC), characterized by 3 features: thrombocytopenia, mechanical hemolytic anemia with schistocytosis, and renal injury (*1*). HUS primarily affects children; incidence in children in France is 1 case/100,000 person-years, 10 times higher than in adults (*2*,*3*). In France, most HUS cases are sporadic, but several outbreaks are reported each year. Those outbreaks are identified through the national pediatric nephrologists’ surveillance network, which declares HUS cases to Santé Publique France, and through sequencing of all STEC isolates from HUS cases referred to the associated National Reference Center (NRC) for *E. coli.*

In France, STEC strains causing HUS mainly belong to serogroups O26, O80, and O157 and, to a lesser extent, to O121, O145, O55, and O103 (*4*). Most of those strains harbor genes encoding Shiga toxin type 2 and notably the 2a or 2d subtypes, which are carried on a prophage. They also contain a chromosomal pathogenicity island, the locus enterocyte effacement (LEE), which mediates intimate adhesion to epithelial cells and is characterized by the presence of the *eae* gene, encoding the intimin protein. Some strains may lack the gene *eae* and are therefore classified as LEE-negative STEC. In addition, the gene encoding enterohemolysin (*ehxA*) is frequently present among STEC (*1*).

In January 2025, the NRC for *E. coli* identified several samples positive for a LEE-negative STEC isolate of an unusual serogroup that harbored *stx*2 alone, mostly from elderly patients hospitalized with HUS in mainland France. Surprisingly, no pediatric HUS cases were reported with that strain. Whole-genome sequencing confirmed the clonality of all isolates, indicating an outbreak of HUS caused by the STEC strain. 

The aim of our study was to describe the outbreak and the LEE-negative STEC strain responsible for severe HUS cases in elderly patients in France with 2 additional cases in Europe. We intended to understand the particular virulence of the strain and the reasons there were no pediatric cases.

## Materials and Methods

### Diagnosis of STEC Infections

The NRC for *E. coli* receives fecal samples collected across France, from patients suspected of HUS or experiencing hemorrhagic diarrhea at any age, as well as from at-risk populations with diarrhea. STEC diagnosis at the NRC is performed by DNA extraction using the Elite Ingenius instrument (Elitech, https://www.elitechgroup.com), followed by PCR with the RIDA GENE *E. coli* Stool Panel (R Biopharm, https://clinical.r-biopharm.com), which enables detection of *stx1, stx2*, and *eae* genes.

We inoculated positive samples onto selective culture media, including CHROMagar STEC (CHROMagar, https://www.chromagar.com), MacConkey CT (bioMérieux, https://www.biomerieux.com), and chromogenic medium chromID CPSO (bioMérieux). Chromogenic medium enables direct identification of *E. coli* when selective media are negative. We identified and confirmed colonies of STEC using the eazyplex EHEC complete kit (Amplex Diagnostics, https://www.eazyplex.com), which detects *stx1, stx2, eae,* and *ehxA* genes. We tested for 10 major STEC serogroups by in-house multiplex PCR as previously described (*5*). Finally, we conducted antimicrobial susceptibility testing on STEC strains by disk diffusion method, before storing them at –80°C.

### Sequencing

We sequenced all strains at Institut Pasteur (Paris, France) using Illumina technology, as previously described (*6*). For 3 isolates, we performed hybrid assemblies with Unicycler (https://github.com/rrwick/Unicycler) to obtain circularized chromosomes and plasmids, using long-read sequencing with the Native Barcoding Kit 96 V14 (SQK-NBD114.96) on a GridION Flow Cell R10 with MinION (Oxford Nanopore, https://nanoporetech.com), combined with Illumina short-read data generated on a MiniSeq (https://illumina.com). We performed quality control short reads and long reads by fastQC (https://github.com/s-andrews/FastQC) combined with multiQC (https://github.com/MultiQC/MultiQC) for short reads and fastQC combined with nanoQC (https://github.com/wdecoster/nanoQC) for long reads.

### Bioinformatics Analysis

We determined the isolate’s phylogroup using Clermontyping (http://clermontyping.iame-research.center). We obtained sequence type (ST) and hierarchical clustering (HC), notably HC5, from Enterobase (https://enterobase.warwick.ac.uk). We performed chromosome and plasmid annotations, including the search for potential virulence genes, with Prokka version 1.14.6 (https://github.com/tseemann/prokka) using a LA database (https://github.com/Kevi84LA/LA-database/blob/main/LA_database.faa). The LA database was built by compiling an updated set of reviewed bacterial entries from UniProt (https://www.uniprot.org), gene sets from the Center for Genomic Epidemiology (https://www.genomicepidemiology.org), the virulence factor database (https://www.mgc.ac.cn/VFs), and in-house sources as previously described (*7*), translated into amino acid format. To avoid redundancy across the multiple databases, we used CD-HIT version 4.8.1 (https://github.com/weizhongli/cdhit) to cluster identical genes into a single representative sequence with 100% identity and 100% coverage.

We performed resistance gene search using Abricate tool on Resfinder database (*8*,*9*). We used PlasmidFinder (https://cge.food.dtu.dk/services/PlasmidFinder) and pMLST (https://cge.food.dtu.dk/services/pMLST) (*10*) for plasmid characterization and performed plasmid alignment using BRIG version 1.0.0 (https://github.com/happykhan/BRIG).

We performed phylogenic analysis with IQ-Tree version 2.4.0 (https://iqtree.github.io); we based the analysis on core-genome alignment of isolates produced by Panaroo version 1.5.2 (https://github.com/gtonkinhill/panaroo). We identified single-nucleotide polymorphisms (SNPs) on the basis of core-genome alignment using pairsnp (https://github.com/gtonkinhill/pairsnp).

### Serotyping

Because the O serogroup deduced from the *wzx* and *wzy* sequences by Enterobase could not distinguish between O17/O44/O73/O77/O106, defining the O77-group (O77 g) as previously described (*11*), we further investigated the O serogroup and H serogroup by phenotypic method at the NRC for *E. coli* in Spain. We determined O and H antigens using the method previously described (*12*); all available O (O1 to O181) antiserums were produced in the Laboratorio de Referencia de *E*. *coli*—University of Santiago de Compostela (Lugo, Spain). Finally, we used the K1/*Neisseria meningitidis* B antiserum (Bio-Rad Laboratories, https://www.bio-rad.com) to characterize the capsular serogroup.

### Specific PCR

To identify patients potentially infected with the outbreak strain (i.e., patients with fecal PCR positive for *stx2* and negative for *eae* during the outbreak period but without a cultured STEC isolate), we developed an in-house quadruplex PCR targeting specific regions. We designed 2 primer pairs to target the large virulence plasmid characteristic of the outbreak strain: the first pair amplifies a 285-bp fragment located between 2 open-reading frames encoding phytase/esterase activity and a putative thiol peroxidase (*tpx* gene), and the second pair amplifies a 163-bp fragment of an open-reading frame encoding a putative thermolabile enterotoxin (*elt* chain A). We designed 2 additional primer pairs to target chromosomal genes, the *wzy* gene encoding the O77-group and the *neuS* K92 capsule gene (Appendix).

### Case Definitions

We classified patients with HUS, diarrhea, or both in whom the outbreak strain was isolated from stool sample as confirmed cases. We classified those patients without the outbreak strain but with a positive quadruplex PCR in stool sample as probable cases. We classified those patients without isolation of the outbreak strain and showing only the chromosomal (plasmid markers negative) on quadruplex PCR in their sample as possible cases.

## Results

### Outbreak Cases 

**Figure 1:**
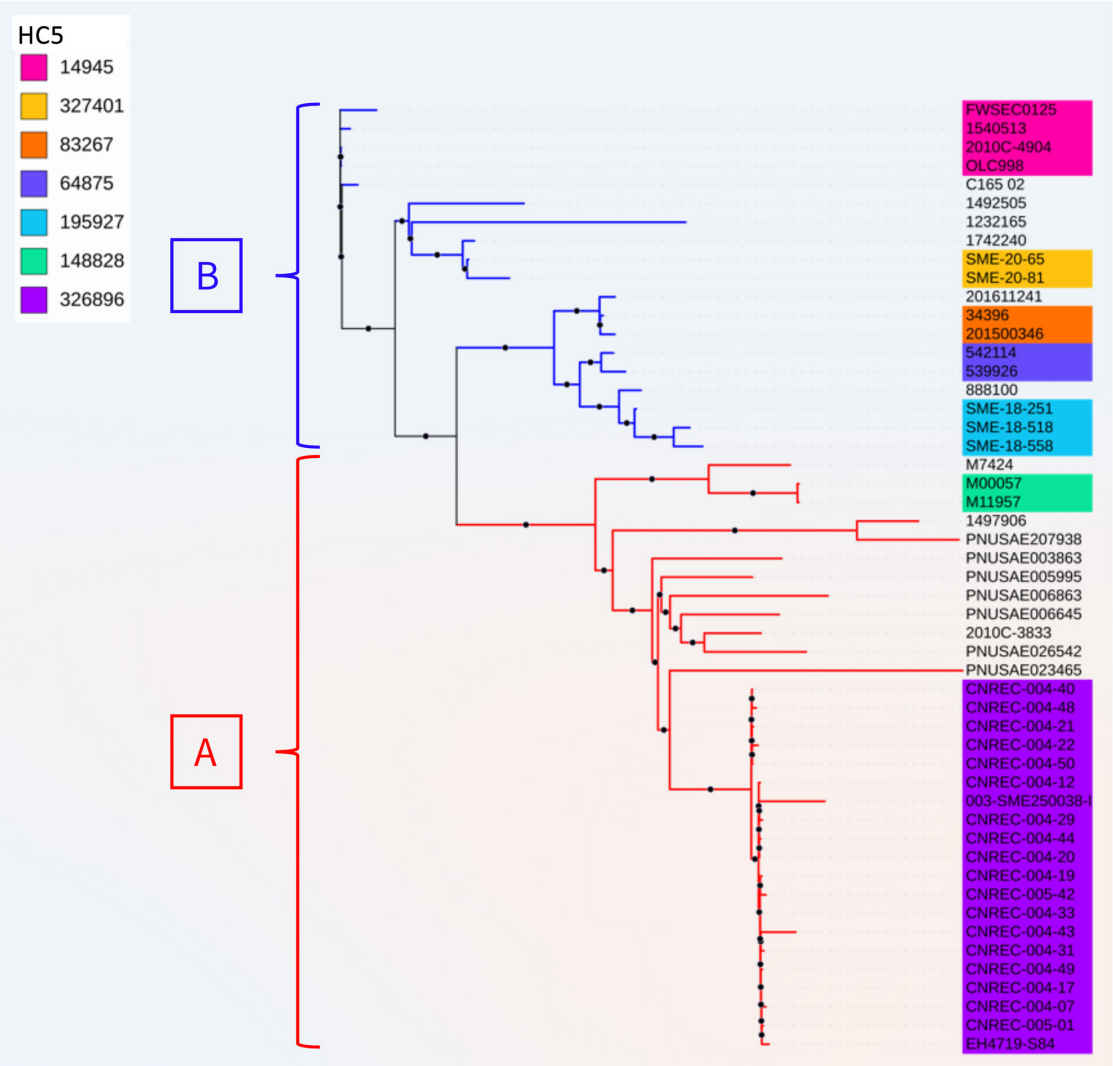
Unrooted phylogenetic tree based on core genome alignment of 51 Shiga toxin–producing *Escherichia coli* isolates from study of hemolytic uremic syndrome outbreak in adults and Shiga toxin–producing *Escherichia coli* negative for locus of enterocyte effacement, France, 2025. Shown are the France 2025 outbreak strain (HC5 326896, in purple) and other strains with the same core-genome multilocus sequence typing HC200 2073 group identified in EnteroBase and GenBank (Appendix Table 1). Isolates are divided into 2 main groups, A and B, in accordance with their phylogenetic distribution. Only HC5 comprising >2 genomes are colored. Figures 1–3 are combined in Appendix Figure 1.

**Figure 2:**
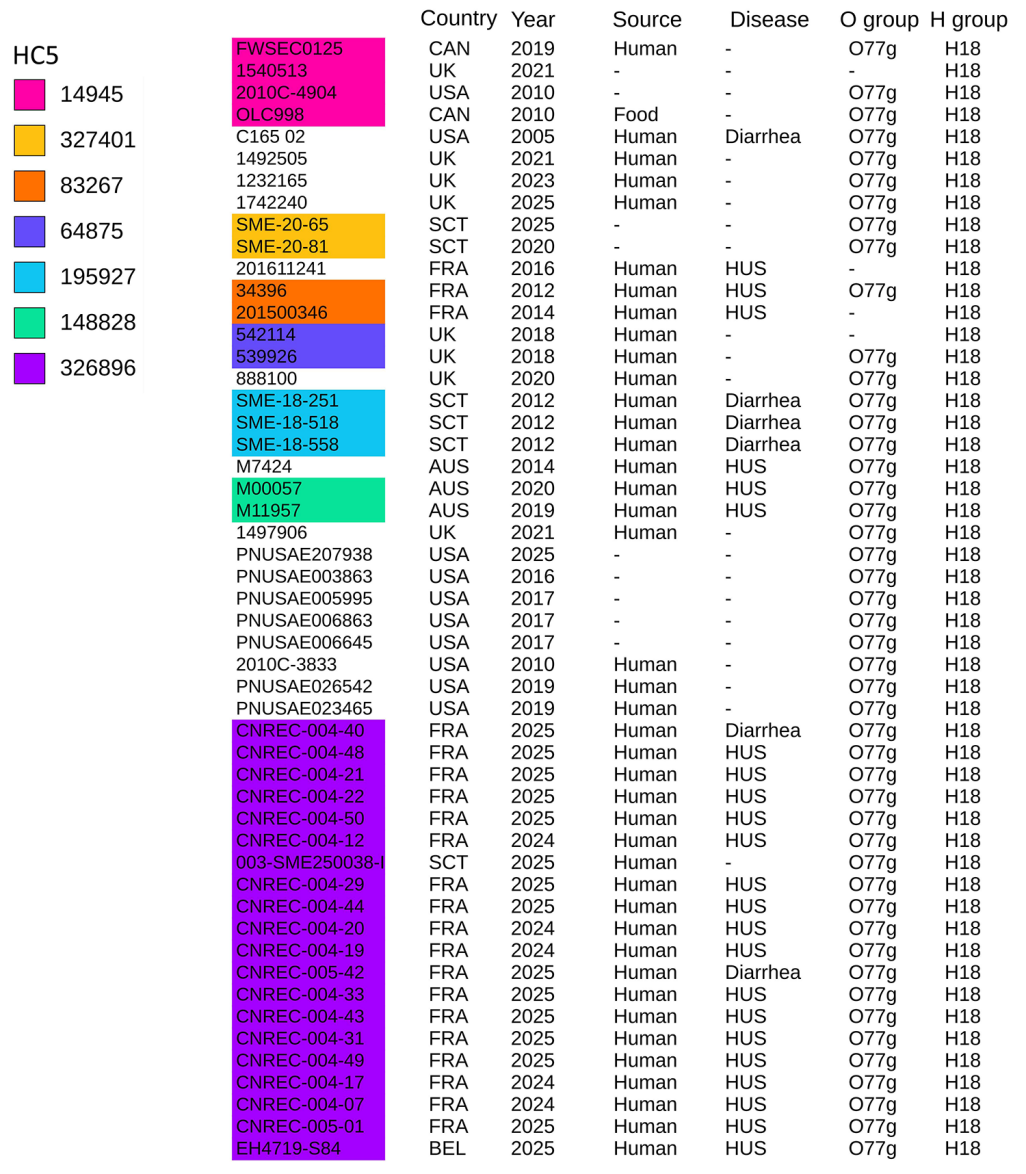
Origins and serotypes of 51 Shiga toxin–producing *Escherichia coli* isolates from study of HUS in adults and Shiga toxin–producing *Escherichia coli* negative for locus of enterocyte effacement, France, 2025. Shown are the outbreak strain (HC5 326896, in purple) and other strains with the same core-genome multilocus sequence typing HC200 2073 group identified in EnteroBase and GenBank (Appendix Table 1). Only HC5 comprising >2 genomes are colored. Country of origin, year of isolation, source, disease, and O and H serotypes are indicated; O77 g denotes serogroup from the O77 group. Dash indicates the serotype is not available. AUS, Australia; BEL, Belgium; CAN, Canada; FRA, France; HUS, hemolytic uremic syndrome; SCT, Scotland; UK, United Kingdom; USA, United States. Figures 1–3 are combined in Appendix Figure 1.

**Figure 3:**
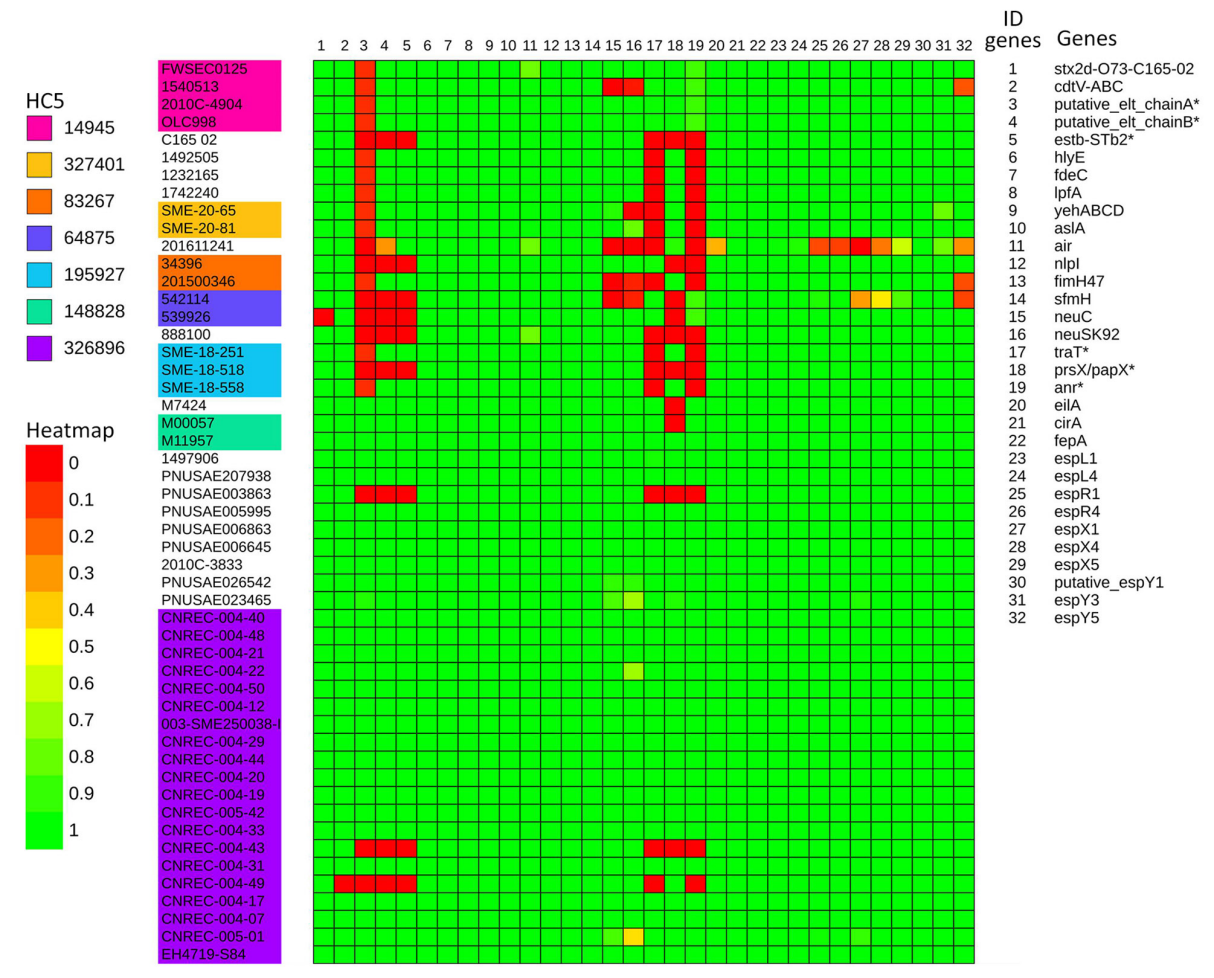
Distribution of genes encoding major virulence factors of 51 Shiga toxin–producing *Escherichia coli* isolates from study of hemolytic uremic syndrome outbreak in adults and Shiga toxin–producing *Escherichia coli* negative for locus of enterocyte effacement, France, 2025. Shown are the France 2025 outbreak strain (HC5 326896, in purple) and other strains with the same core-genome multilocus sequence typing HC200 2073 group identified in EnteroBase and GenBank (Appendix Table 1). Heatmap assembled from phylogenic tree using ITOL tool (https://itol.embl.de), based on gene sequences of reference isolate CNREC_004-7. The score represents the combination of percentage coverage and percentage nucleotide identity. Asterisk indicates genes located on the large plasmid (134 kb). Figures 1–3 are combined in Appendix Figure 1.

At the end of December, the NRC for *E. coli* identified 4 adult HUS cases, caused by a LEE-negative STEC that did not belong to the 10 usual major serogroups determined by routine PCR and harbored that *stx2* but not *eae* or *ehxA* genes. Of note, isolates failed to grow on selective STEC media and were recovered only on CPSO plates. Illumina sequencing confirmed that the outbreak was caused by a unique strain, O77 g:H18, belonging to ST69 of phylogroup D, harboring the *stx2d* subtype and lacking *eae* and *ehxA*. The strain belonged to the novel cluster at the HC5 level 326896, consistent with the core genome multilocus sequence typing–based hierarchical clustering scheme proposed by EnteroBase; outbreak-related isolates presented allelic distances <5. We further confirmed isolate similarity by a core-genome SNP phylogenetic analysis with a SNP median of 4 (range 1–29) (Figures 1, 2, 3; Appendix Figure 1).

**Figure 4:**
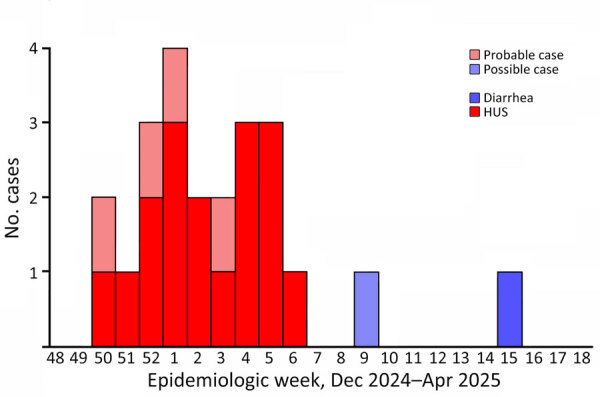
Epidemic curve of confirmed, probable, and possible cases of Shiga toxin–producing *Escherichia coli* infection caused by the outbreak strain (HC5 326896) from study of HUS outbreak in adults and Shiga toxin–producing *Escherichia coli* negative for locus of enterocyte effacement, France, 2025. HUS, hemolytic uremic syndrome.

In total, we identified 18 confirmed cases of STEC infection with the outbreak strain. The cases occurred during December 15, 2024–April 11, 2025 (2024 epidemiologic week 50 through 2025 epidemiologic week 15) (Figure 4); most cases were reported during December 15, 2024–February 3, 2025 (2024 epidemiologic week 50 through 2025 epidemiologic week 6). Cases were distributed nationwide with no clusters (Appendix Figure 2).

**Figure 5:**
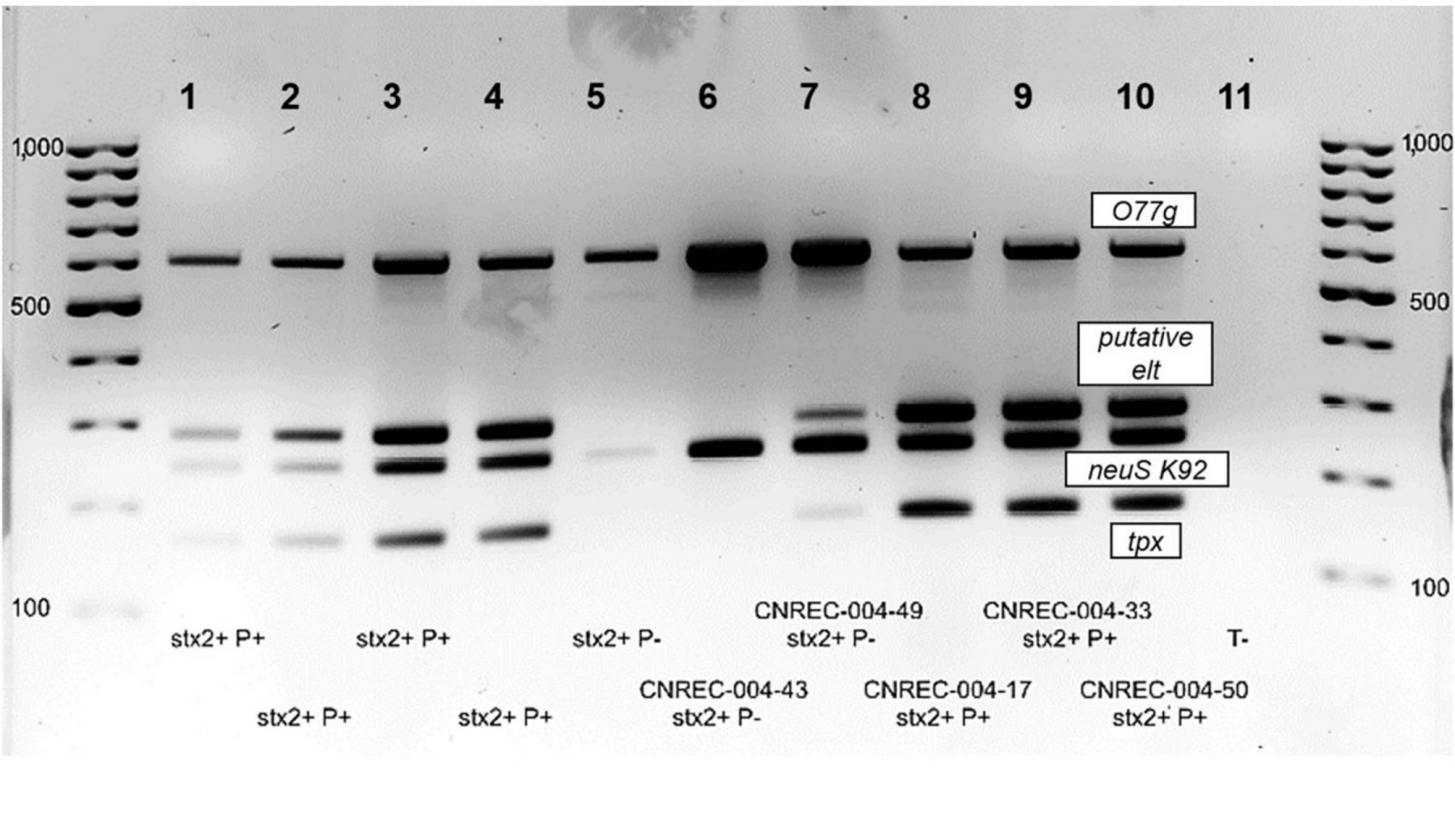
Quadruplex PCR results from study of hemolytic uremic syndrome outbreak in adults and Shiga toxin–producing *Escherichia coli* negative for locus of enterocyte effacement, France, 2025. We performed quadruplex PCR on fecal extracts targeting 2 chromosomic genes: *O77 g* (*wzy*) (609-bp) and *neuS* K92 (247-bp), and 2 loci on the 134 kb plasmid: *elt* (163-bp) and the phytase-*tpx* region (285-bp). Wells 1 to 4, fecal samples (*stx2*+ but culture negative) positive for both chromosomal and plasmid targets (P+) defining probable cases. Well 5, fecal samples positive only for chromosomal targets defining possible case. Well 6, fecal specimen from patient with isolate CNREC-004–43, which had lost its 134kb plasmid probably in vivo. Well 7, specimen from patient with isolate CNREC-004-49, showing plasmid target signals absent in the corresponding isolate DNA, suggesting partial plasmid loss in vitro (Figure 3). Wells 8–10, fecal extracts from patients with outbreak isolates harboring the complete 134kb plasmid (CNREC-004-17, -33 and -50). Well 11, negative control.

To explore the potential extent of the outbreak in patients without STEC isolation, we implemented an in-house specific multiplex PCR. During the outbreak period, extended to 1 month before and 1 month after, we found 38 patients (3 experiencing HUS symptoms and 35 with diarrhea) from whom no STEC was isolated from their fecal cultures to be positive for *stx2* and negative for *eae*. We validated our in-house quadruplex PCR, targeting 2 chromosomal genes and 2 genes present in the 134-kb plasmid, for specificity (Appendix Figure 3) and then applied directly to fecal DNA extracts to identify additional cases. We classified 4 additional patients who tested positive for all 4 target genes as probable cases; 1 patient, whose sample was positive for 2 chromosomal targets, was classified as a possible case (Figures 4, 5).

All patients were adults; median age was 72.1 (interquartile range 66.7–81.8; range 34.3–89.4) years, and female/male ratio was 0.56. Among the 18 confirmed cases and 5 probable and possible cases, 17 (94%) confirmed and 4 (80%) probable and possible, had HUS. Among the 23 case-patients, 13 experienced diarrhea; 4 of those had bloody diarrhea. Three (13%) patients died. We expect to publish clinical details of patients and their outcomes in the future.

### Investigations and Control Measures

Epidemiologic and traceback investigations, including food questionnaires and supermarket loyalty card records, indicated that by the end of January 2025 a total of 15/17 (88%) confirmed and 2/4 probable case-patients had consumed the same brand of raw cow’s-milk cheese. We excluded 2 probable and 1 possible cases, as well as 1 case confirmed late in our investigation on April 11. We identified the outbreak strain in a cheese sample collected from a patient’s refrigerator. As of January 24, 2025, authorities initiated recall and withdrawal of the suspected cheese produced starting November 12, 2024. In February 2025, we identified 2 additional cases through Enterobase; both had STEC isolates belonging to the same HC5 cluster (Figure 1). One case-patient in Belgium experienced HUS; the patient had not consumed the implicated cheese. The other case was in Scotland; the patient’s clinical condition and exposure data were unknown.

### Molecular Characterization of Outbreak Strain

We submitted the 18 isolates from France to short-read whole-genome sequencing; they belonged to ST69 (phylogroup D) and HC5 326896. The *rfb* gene cluster sequence did not allow discrimination between O17/44/73/77/106 antigens, defining the O77-group (O77 g) described previously (*11*), whereas the *fliC* gene corresponded to H18. Agglutination with O antiserum specific to those 5 antigens was negative; therefore, the outbreak strain was designated O77 g:H18. All isolates harbored the *stx2d*-O73-C165-02 variant and were negative for *eae* and *ehxA*, as expected. We long-read sequenced 3 isolates (CNREC-004-07, CNREC-004-31, CNREC-004-43), revealing a chromosome of ≈4,934 kb, a plasmid of ≈96 kb, and, in the first 2 isolates, an additional plasmid of ≈134 kb (Appendix Table 2). The 134-kb plasmid was an IncF plasmid with a complete *tra* operon. The pMLST tool could not precisely identify the F replicon ST (F-:A17*:B24–79*, in which the asterisks indicate new alleles closely related to A17 and B24/B79). The 96-kb plasmid was an IncY plasmid, lacking a *tra* operon (Appendix Table 2).

### Putative Virulence Factors

**Figure 6:**
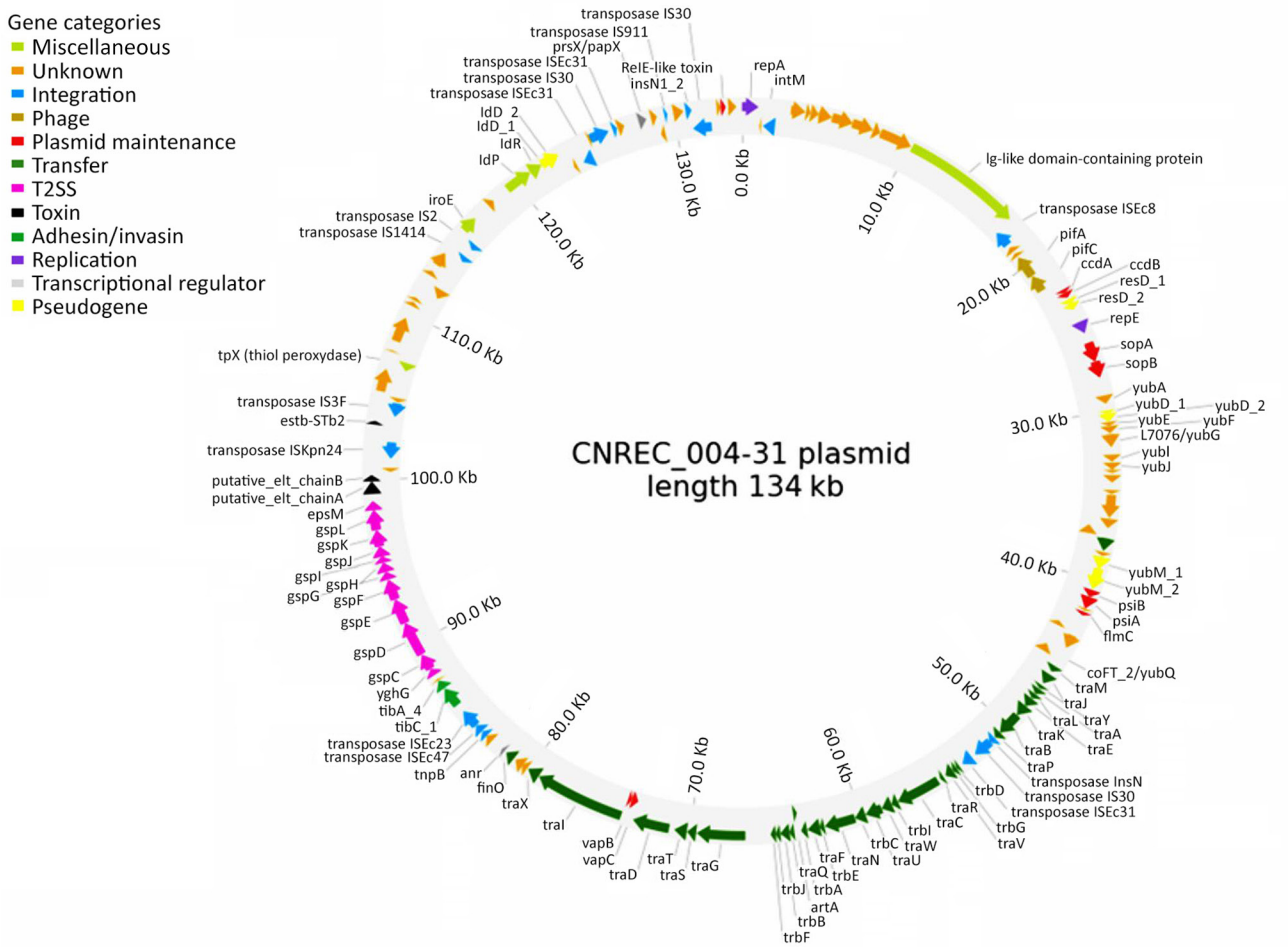
Representation of the 134-kb plasmid from isolate CNREC-004-31 in study of hemolytic uremic syndrome outbreak in adults and Shiga toxin–producing *Escherichia coli* negative for locus of enterocyte effacement, France, 2025. Annotated genes were categorized by function.

We identified several putative virulence factors (Figure 3), some of which were located on the 134-kb plasmid (Figure 6), whereas we detected no known virulence factors on the 96-kb plasmid (data not shown). In addition to the main virulence factor *stx2d*-O73-C165-02, we identified several toxin-encoding genes, including *cdt*-VABC (cytolethal distending toxin subtype V), a putative *elt*AB (heat-labile enterotoxin II), *estb-*STb2 (heat stable enteroxin), and *hlyE* (avian hemolysin). We also detected genes encoding adhesion or invasion factors, particularly *fdeC*, *lpfA*, *yehABCD*, *aslA*, *air*, *fimH*47, and *nlpI*. Furthermore, protectin-encoding genes such as *traT* and *neuABCDES* were present. Other virulence genes with diverse putative functions included the transcriptional regulators *prsX/papX* and *anr*, as well as *eilA*, a transcriptional regulator of the enteropathogenic *E. coli* type III secretion system 2, characteristic of ST69 strains (*13*), and several type III secretion system effectors. None of the 18 outbreak isolates we sequenced carry the *iha* and *aggR* genes, which have been described in LEE-negative STEC (*14*).

The detection of the *neuC* gene suggested the presence of a K1 capsule. However, agglutination with K1-specific antiserum was negative for all isolates except CNREC-004-43. Sequence analysis of the entire *neu* gene cluster showed high homology across all genes (>98%), except for *neuS* (87%). BLAST analysis of the *neuS* sequence revealed 99.92% identity with the K92 *neuS* gene (Appendix Figure 4). Therefore, we considered the outbreak strain serotype O77 g:K92:H18. In isolate CNREC-004-43, which tested K1-positive, we identified a C154A substitution in *neuS* that led to an H52N amino acid change (Appendix Figure 4). That mutation is known to be sufficient to convert a K92 capsule into K1 capsule, explaining the phenotype observed in this isolate (*15*). The outbreak strain carried no antimicrobial resistance genes, confirmed by phenotypic tests. Only a rarely reported parC.S57T mutation was identified, with no effect on quinolone susceptibility.

### Similar Strains Worldwide

To determine whether other isolates closely related to our outbreak strain exist worldwide, we searched Enterobase for isolates with the same HC200 2073. We identified 30 such isolates (Figure 1), spanning 3 continents. Moreover, we found in GenBank the strain C165-02, linked to *stx2d*-O73-C165-02, which is the oldest strain we reported here, isolated in 2005 in Maryland from a patient with bloody diarrhea (*16*). Almost all isolates were of human origin, but clinical information, including patient age, was missing for most. Several cases were associated with HUS, consistent with the virulence of the outbreak strain. All those isolates are ST69 and have common features, including the *stx2d*-O73-C165-02 variant (except 1), *cdtV* (except 1 outbreak isolate), *hlyE*, and multiple adhesion and miscellaneous genes. We divided the population into 2 main groups, A and B, on the basis of phylogenetic analysis and gene distribution (Figure 1).

Group A (n = 32), which includes our outbreak isolates and, of note, 3 circularized strains from Australia, carried nearly all of the putative virulence factors described, with the exception of 3 isolates. The 3 exceptions were characterized by partial or complete loss of 134 kb plasmid-borne genes (putative *eltAB*, *STb*, *traT*, *prsX/papX*, and *anr*) (Figures 1, 2, 3). To further investigate the plasmid content of all HC200 2073 strains, we performed multi-alignments using BRIG. As previously indicated, CNREC-004-43, one of our long-read sequenced isolates, had completely lost the 134-kb plasmid, whereas CNREC-004-49 and PNUSAE003863 had deletions encompassing large portions of the plasmid (Appendix Figure 5, panels A, B). Of interest, our in-house quadruplex PCR detected plasmid target signals in stool samples containing CNREC-004-49, suggesting in vitro plasmid loss. Altogether, those findings indicate that the 134-kb plasmid is relatively unstable and can be lost either partially or entirely, even in the context of an outbreak and over a short time. In contrast, in group A, only 3 isolates outside the outbreak carried the 96-kb plasmid (Appendix Figure 6, panels A, B).

Group B isolates (n = 19) were all characterized by the loss of >1 plasmidborne gene (putative *eltAB*, *STb*, *traT*, *prsX/papX*, and *anr*) (Figures 1, 2, 3). Of note, although isolates were from different countries in Europe or North America, none carried the putative *elt* chain A gene. Only 4 group B isolates harbored an almost complete 134-kb plasmid, and all shared the same HC5 14945 (Figure 1; Appendix Figure 5, panel C). Nine isolates retained a large portion of the 134-kb plasmid but lacked the *tra* operon (Appendix Figure 5, panel C). Ten isolates carried the 96-kb plasmid, including the oldest strain, C165-02 (Appendix Figure 6, panel C). Six group B isolates might not produce a K92 capsule (Figure 1), because *neuC* and *neuS* K92 were absent. Sequence alignment of those 6 isolates with the capsular operon of CNREC-004-31 revealed the presence of a complete *kps* operon but high fragmentation of *neu* genes (Appendix Figure 7).

## Discussion

In this study, we describe a LEE-negative STEC/enterotoxigenic (ETEC) *E. coli* strain with several unique features that was linked to a HUS outbreak occurring exclusively in adults. The outbreak strain belonged to ST69, a major ST of phylogroup D. In contrast, most STEC-causing HUS belong to phylogroups B1 or E (*17*). ST69 strains are usually associated with extraintestinal infections (*17*); however, none of our patients experienced systemic infection. Thus, the genetic background of this intestinal pathogenic *E. coli* is highly unusual.

Clinical manifestations associated with infection by the outbreak strain were highly severe; HUS rate was 91%, one of the highest ever reported among STEC outbreaks internationally whether in children (1%–56% of HUS) or adults (3%–24%) (*2*). The predominance of elderly patients, higher than previously reported for adult HUS sporadic cases in France (*18*), partly explains the severity, although only 1 patient, a 72-year-old kidney transplant recipient, was immunocompromised and at higher risk for HUS (*19*).

Several other features of the outbreak strain may have contributed to its severity. First, the toxin Stx2d is strongly associated with HUS in France, both in children and adults (*20*). The specific variant identified in this study, *stx2d*-O73-C165-02, is rare (*21*), was described in 2007 (*22*), and may display higher virulence compared with other *stx2d* variants (*23*). Second, the outbreak strain harbors additional toxin genes that could have exacerbated disease. Those genes include the putative heat labile enterotoxin II (*eltAB*) and the heat-stable enterotoxin type b (*STb*), suggesting a hybrid STEC/ETEC pathotype. Emerging STEC/ETEC hybrids have been increasingly reported worldwide, isolated from animals, food, and humans in the context of diarrhea or HUS (*24*–*26*). Finally, the strain encodes the toxin CDT-V, previously identified in other *eae*–non-O157 STEC, such as O91 (*14*), which might also have contributed to its pathogenicity. Indeed, CDT-V is a genotoxin and cyclomodulin that causes DNA damage, cell cycle arrest, and ultimately the death of human microvascular endothelial cells.

Our outbreak was also characterized by infections occurring exclusively in adults. STEC infections, particularly HUS, are ≈10 times less frequent in adults than in children (*2*,*3*). However, few outbreaks exclusively affecting adults have been reported internationally, all caused by O157:H7 STEC, and most of them occurred in nursing homes, which explains the population affected (*2*,*27*–*29*).

Several hypotheses may explain the unusual epidemiology. First, in France, several widely publicized HUS outbreaks in children during 2014–2024, many linked to raw milk cheese, have likely increased awareness of the illness and at-risk foods. National recommendations on STEC prevention explicitly include advising against the consumption of raw milk cheese in children <5 years of age. Second, and perhaps more intriguingly in this outbreak, protection in children could be linked to the expression of the rare K92 capsule by the outbreak strain. The K92 capsule, structurally related to the K1 capsule, is composed of a homopolymer of N-acetyl-neuraminic acid with alternating, α2–8 and α2–9 linkages. Of note, the meningococcal C capsule (α2–9 linkages) and meningococcal B capsule (α2–8 linkages) share structural similarities. There is therefore a homology between those capsules; experimental cross-immunogenicity between *E. coli* K92 and meningococcus C has already been demonstrated (*15*,*30*). Thus, mandatory meningococcal C vaccination of infants in France since 2018, with a high pediatric population coverage of 88.6% in 2024 (*31*), might have conferred cross-protection against K92 *E. coli* infections in children. However, we note that we did not demonstrate the expression of the K92 capsule because K92 antiserum was unavailable. Finally, our in-house quadruplex PCR showed that we missed few cases by culture, and the assay could serve to detect the reemergence of this strain. It could also help ecologic niche investigations of the unusual STEC/ETEC strain.

Interrogation of international databases identified 31 additional stains, all belonging to HC200 2073, that were closely related to our outbreak strain and were distributed across 3 continents. The earliest reported isolate, C165-02, was recorded in 2005 in Maryland, USA, from a patient with bloody diarrhea; its name is used for the *stxd2*-O73-C165-02 variants. Almost all the HC200 2073 strains have been isolated from humans. Of note, some shared the same HC5 and were potentially involved in small outbreaks (Figures 1, 2). Those findings highlight that the *E. coli* hybrid pathotype ST69, carrying the *stx2d*-O73-C165-02 variant, has been spreading worldwide and warrants close monitoring, given its outbreak potential and severity.

In conclusion, we characterized a singular emerging hybrid STEC/ETEC pathotype responsible for a severe HUS outbreak exclusively in elderly patients. The identification of genetically related isolates worldwide since 2005 suggests recent global emergence and underscores the need for strengthened epidemiologic surveillance to track its spread. Just before the outbreak, HUS surveillance in France for adults was formally implemented to better capture the characteristics of STEC infections in this population. While such outbreaks remain rare, occurrence of this outbreak highlights the severe health impact and outbreak potential of STEC in adults and the risk presented by atypical emerging strains. Our in-house PCR can help detect outbreaks, investigate their sources, and identify reservoirs. Finally, the possible protective effect of meningococcal C vaccination could represent an unexpected defense against this clone.

Appendix Additional information about a hemolytic uremic syndrome outbreak in adults and Shiga toxin–producing *Escherichia coli*, France, 2025.
