# Supplementary material for: Hemolytic Uremic Syndrome Outbreak in Adults and Shiga Toxin–Producing Escherichia coli Negative for Locus of Enterocyte Effacement, France, 2025
Source: Emerg Infect Dis. 2026 Apr;32(4):592–602. doi: 10.3201/eid3204.251417 (PMC13094856; doi:10.3201/eid3204.251417)
Supplement: Appendix — Additional information about a hemolytic uremic syndrome outbreak in adults and Shiga toxin–producing Escherichia coli, France, 2025. [file 25-1417-Techapp-s1.pdf]

Article DOI: https://doi.org/10.3201/eid3204.251417

EID cannot ensure accessibility for supplementary materials supplied by authors. Readers who have difficulty accessing supplementary content should contact the authors for assistance.

Hemolytic Uremic Syndrome Outbreak in Adults and Shiga Toxin–Producing *Escherichia coli* Negative for Locus of Enterocyte Effacement, France, 2025

Appendix

Specific In-House Quadruplex PCR to Detect Outbreak Strain from Stool Samples

To identify patients potentially infected with the outbreak strain (i.e., patients with stool PCR *stx2*+/*eae*- during the outbreak period but without a cultured STEC isolate), an in-house quadruplex PCR was developed targeting specific regions. Two primer pairs were designed to target the large virulence plasmid characteristic of the outbreak strain (see Results): the first pair, STEC69-F (5′-CTGGTGTAGTCCAATCAGGCAT-3′) / STEC69-R (5′-TGACTTCGCTGGGAACTCTTAC-3′), amplifies a 285-bp fragment located between two ORFs encoding phytase/esterase activity and a putative thiol peroxidase (*tpx* gene); the second pair, LT2A69-F (5′-TGGTCAGAACATGTTTGGTGGA-3′) / LT2A69-R (5′-TCCGATAACCTGAGACCATGGA-3′), amplifies a 163-bp fragment of an ORF encoding a putative thermolabile enterotoxin (*elt* chain A). Two additional primer pairs were designed to target chromosomal genes: a pair which amplify a 609-bp fragment, containing the *wzy* gene encoding the O77-group (O17/O77/O44/O106) antigen polymerase, which is rare in the *E.coli* species: O77 g-F (5′-TTCCATCCTTTACTTGCAAACAA-3′) and O77 g-R (5′-ACCACAGCGGGATGAAGTTGAT-3′), and another pair, amplifying a 247-bp fragment, encoding the uncommon *neuS* K92 capsule gene variant identified in the outbreak strains: *neuS*-K92-F (5′-GCTGAAGAGCTACTATCACCGT-3′) and *neuS*-K92-R (5′-AAGCGAAAATGTATTTGGCTCT-3′).

The specificity of the quadruplex PCR was first verified *in silico* using on the GenBank NCBI database, and then in vitro on stool samples culture-positive for the outbreak strain, which

served as positive control. As negative controls, several stool samples with a positive culture for a STEC isolate different than the epidemic strain, were tested.

Quadruplex PCR was performed in a single-tube multiplex reaction (50µl) using the Qiagen® multiplex PCR kit (Qiagen®, Valencia, CA) with Q solution, 5µL of DNA extract (stool lysates obtained after boiling), and primers at a final concentration of 0.2 µM. Cycling conditions consisted of an initial denaturation at 95°C (15 min), followed by 30 cycles of denaturation at 94°C for 30 s, annealing at 55°C for 90 s, and extension at 72°C for 90 s, with a final extension at 72°C for 10 min. Amplicons were resolved by electrophoresis on a 2% standard agarose gel stained with ethidium bromide for 50 min at 135 V.

Appendix Table 1. Summary of HC200 2073 isolates identified in Enterobase and NCBI, including outbreak isolates, ordered by HC*

Strain (sequencing no.)	Year	Country	Common HC with outbreak strain	Sequencing type	Enterobase barcode	GenBank accession no.
CNREC_004–07 (202500057)	2024	FRA	HC5 326896	Illumina & Nanopore	ESC_PB7223AA	ERR15529900 & JBRWUM000000000
CNREC_004–12 (202500190)	2024	FRA	HC5 326896	Illumina	ESC_PB7659AA	ERR15529901
CNREC_004–17 (202500194)	2024	FRA	HC5 326896	Illumina	ESC_PB7654AA	ERR15529902
CNREC_004–19 (202500196)	2024	FRA	HC5 326896	Illumina	ESC_PB7658AA	ERR15529903
CNREC_004–20 (202500450)	2024	FRA	HC5 326896	Illumina	ESC_PB8919AA	ERR15529904
CNREC_004–21 (202500451)	2025	FRA	HC5 326896	Illumina	ESC_PB8920AA	ERR15529905
CNREC_004–22 (202500453)	2025	FRA	HC5 326896	Illumina	ESC_PB8917AA	ERR15529906
CNREC_004–29 (202500604)	2025	FRA	HC5 326896	Illumina	ESC_QB0282AA	ERR15529907
CNREC_004–31 (202500606)	2025	FRA	HC5 326896	Illumina & Nanopore	ESC_QB0286AA	ERR15529908 & JBRWUL000000000
CNREC_004–33 (202500746)	2025	FRA	HC5 326896	Illumina	ESC_QB1507AA	ERR15529909
CNREC_004–40 (202500887)	2025	FRA	HC5 326896	Illumina	ESC_QB2520AA	ERR15529911
CNREC_004–43 (202500892)	2025	FRA	HC5 326896	Illumina & Nanopore	ESC_QB2522AA	ERR15529912 & JBRWUK000000000
CNREC_004–44 (202500886)	2025	FRA	HC5 326896	Illumina	ESC_QB2521AA	ERR15529910
CNREC_004–48 (202501052)	2025	FRA	HC5 326896	Illumina	ESC_QB3487AA	ERR15529913
CNREC_004–49 (202501053)	2025	FRA	HC5 326896	Illumina	ESC_QB3484AA	ERR15529914
CNREC_004–50 (202501054)	2025	FRA	HC5 326896	Illumina	ESC_QB3485AA	ERR15529915
CNREC_005–01 (202501653)	2025	FRA	HC5 326896	Illumina	ESC_QB8198AA	ERR15529916
CNREC_005–42 (202502735)	2025	FRA	HC5 326896	Illumina	ESC_RB8046AA	ERR15529917
EH4719-S84	2025	BEL	HC5 326896	Illumina	ESC_QB7963AA	DBHFFB000000000
003-SME250038-I	2025	SCT	HC5 326896	Illumina	ESC_PB8936AA	-
PNUSAE026542	2019	USA	HC50 2073	Illumina	ESC_NA4904AA	AARELP01
2010C-3833	2010	USA	HC100 2073	Illumina	ESC_CA8074AA	AATCOA000000000
PNUSAE003863	2016	USA	HC100 2073	Illumina	ESC_EA3290AA	AASYFJ000000000
PNUSAE005995	2017	USA	HC100 2073	Illumina	ESC_FA9267AA	AASQPX000000000
PNUSAE006645	2017	USA	HC100 2073	Illumina	ESC_GA1861AA	AASPQB000000000
PNUSAE006863	2017	USA	HC100 2073	Illumina	ESC_GA4692AA	AASPEX000000000

Strain (sequencing no.)	Year	Country	Common HC with outbreak strain	Sequencing type	Enterobase barcode	GenBank accession no.
M7424	2014	AUS	HC200 2073	Nanopore	ESC_SA7977AA	CP063153 (chromosome)<br>CP063154 (plasmid 1)<br>CP063155 (plasmid 2)<br>CP063156 (plasmid 3)
M11957	2019	AUS	HC200 2073	Nanopore	ESC_SA7976AA	CP061337 (chromosome)<br>CP061338 (plasmid)
M00057	2020	AUS	HC200 2073	Nanopore	ESC_SA7975AA	CP061339 (chromosome)<br>CP061340 (plasmid)
C165–02	2005	USA	HC200 2073	Illumina	-	AFDR00000000
2010C-4904	2010	USA	HC200 2073	Illumina	ESC_BA0743AA	AATIFR000000000
PNUSAE023465	2019	USA	HC200 2073	Illumina	ESC_MA3045AA	AARHWP01
PNUSAE207938	2025	USA	HC200 2073	Illumina	ESC_RB2569AA	ABYPZU000000000
OLC998	2010	CAN	HC200 2073	Illumina	ESC_HA2858AA	NZ_NEMH00000000
FWSEC0125	2019	CAN	HC200 2073	Illumina	ESC_LA1990AA	NZ_RRGQ00000000
SME-18–558	2012	SCT	HC200 2073	Illumina	ESC_PB8560AA	DAHCBK000000000
SME-18–518	2012	SCT	HC200 2073	Illumina	ESC_PB8941AA	DAHBPY000000000
SME-18–251	2012	SCT	HC200 2073	Illumina	ESC_PB8942AA	DAHBNC000000000
SME-20–81	2020	SCT	HC200 2073	Illumina	ESC_PB8559AA	-
SME-20–65	2025	SCT	HC200 2073	Illumina	ESC_QB5887AA	-
34396	2012	FRA	HC200 2073	Illumina	ESC_VA6703AA	SAMN19553806
201500346	2014	FRA	HC200 2073	Illumina	ESC_KA9411AA	-
201611241	2016	FRA	HC200 2073	Illumina	ESC_KA9466AA	-
539926	2018	UK	HC200 2073	Illumina	ESC_IA9706AA	AAVUFB000000000
542114	2018	UK	HC200 2073	Illumina	ESC_JA0670AA	AARXIN000000000
888100	2020	UK	HC200 2073	Illumina	ESC_QA5773AA	AATBQK000000000
1497906	2021	UK	HC200 2073	Illumina	ESC_XA1715AA	ABDIWS000000000
1540513	2021	UK	HC200 2073	Illumina	ESC_XA9798AA	ABEEBJ000000000
1492505	2021	UK	HC200 2073	Illumina	ESC_XA1047AA	ABDDOB000000000
01232165	2023	UK	HC200 2073	Illumina	ESC_KB3959AA	ABSLOE000000000
01742240	2025	UK	HC200 2073	Illumina	ESC_RB9116AA	SAMN48561681

*Countries: AUS, Australia; BEL, Belgium; CAN, Canada; FRA, France; SCT, Scotland; UK, United Kingdom; USA, United States of America.

Appendix Table 2. Genomic characteristics of three outbreak isolates submitted to long-read sequencing*

	Isolates								
	CNREC-004–07			CNREC-004–31			CNREC-004–43		
Characteristic	K	Large P	Small P	K	Large P	Small P	K	Large P	Small P
Size, pb	4936116	134007	94697	4934885	133975	96111	4933657	-	96111
Inc group		IncF	IncY		IncF	IncY		-	IncY
Replicon seq. type		F-:A17*:B24–79*	-		F-:A17*:B24–79*	-		-	-
tra gene cluster		Present	Absent		Present	Absent		-	Absent

K chromosome; P, plasmid; F-:A17:B24–79*, asterisks indicate new alleles close to A17 and B24/B79.

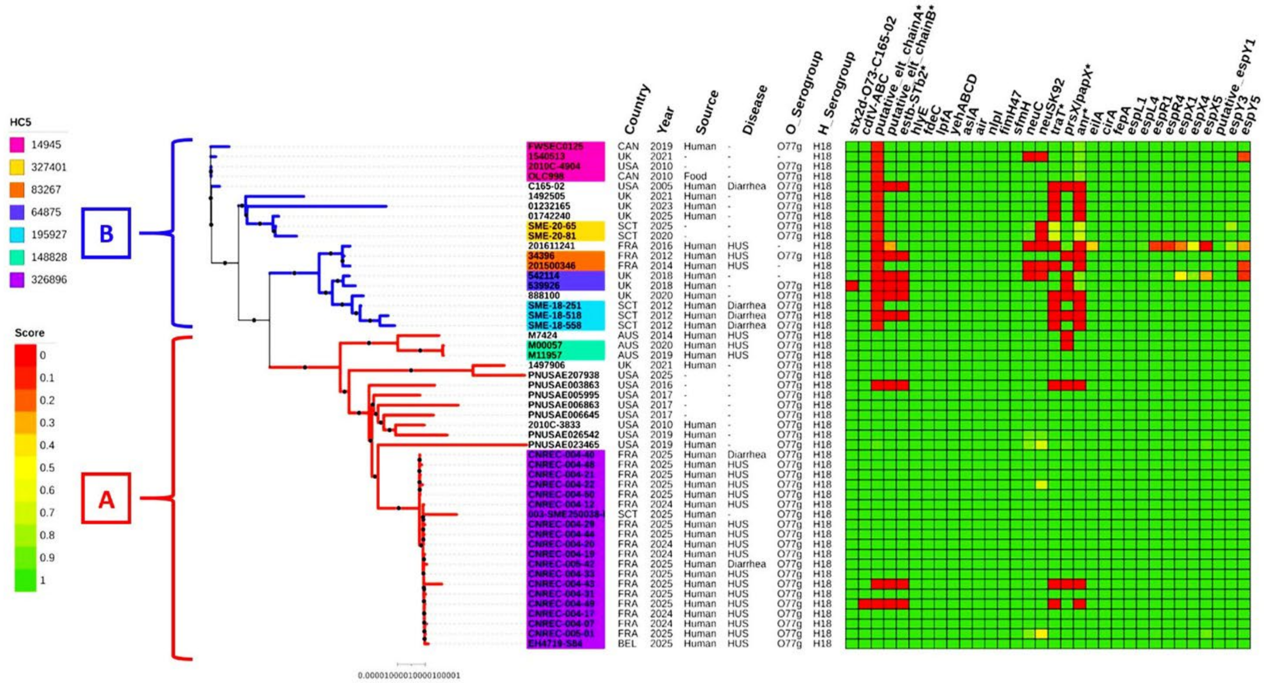

Appendix Figure 1. Unrooted phylogenetic tree based on core genome alignment of 51 isolates, including the outbreak strain (HC5 326896, in purple) and other strains with the same cgMLST HC200 2073 group identified in EnteroBase and NCBI (accession numbers listed in Appendix Table 1). Isolates are divided in two main group A and B according to their phylogenetic distribution. Genome names are colored on their HC5. Only HC5 comprising at least two genomes are colored. Country of origin (FRA: France; BEL: Belgium; SCT: Scotland; UK: United Kingdom; AUS: Australia; USA: United State of America), year of isolation, source, disease, and serotype are indicated (O77g denotes serogroup from the O77-group; (-): not available). The distribution of major virulence factors is shown as a heatmap, assembled with the phylogenic tree using ITOL tool (https://itol.embl.de), based on gene sequences of isolate CNREC_004-7 used as reference. The score represents the combination of % coverage and % nucleotide identity. Genes marked with a star are located on the large plasmid (134-kb).

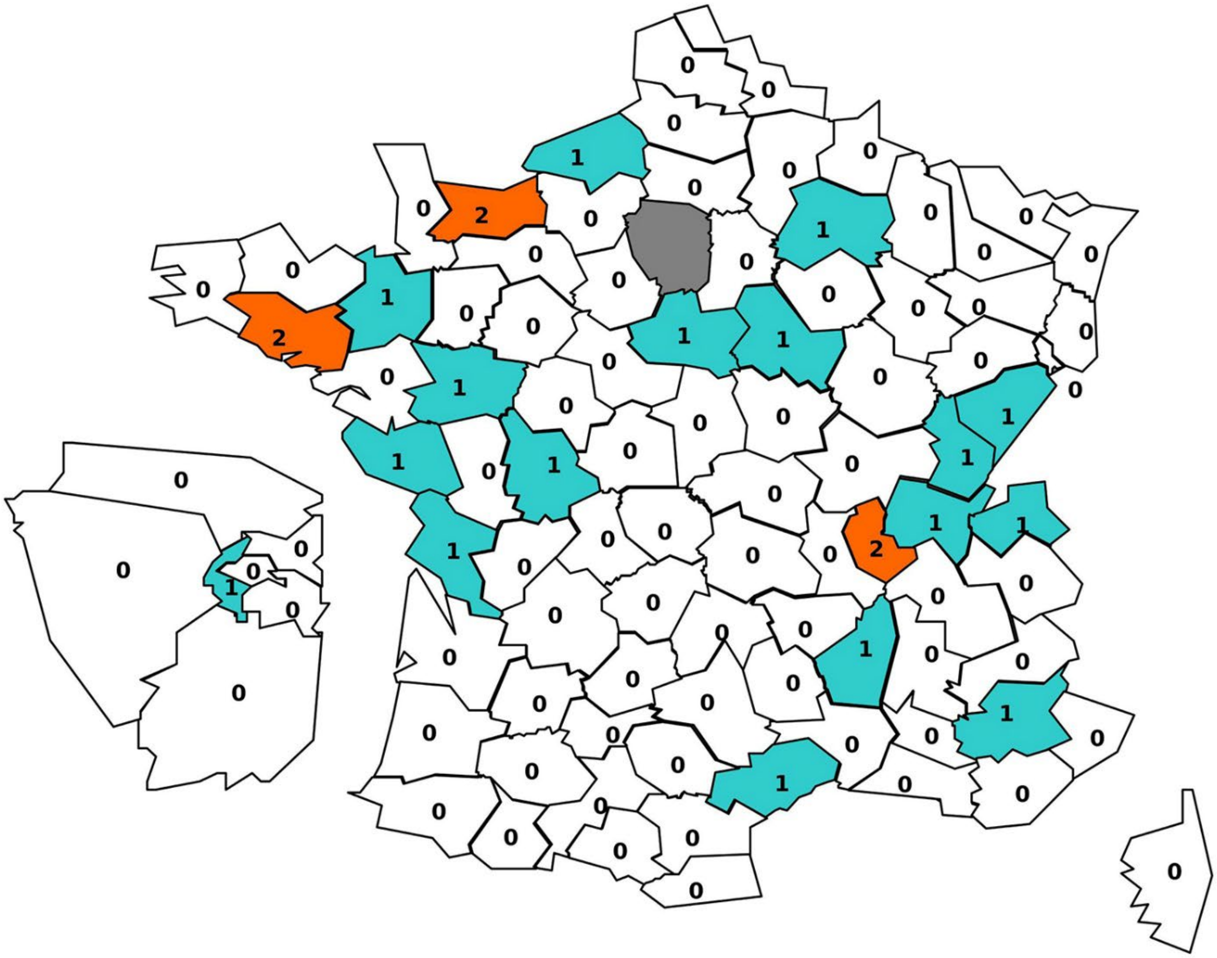

Appendix Figure 2. Geographic repartition of the 23 outbreak cases (confirmed, probable and possible) in mainland France. Blue color: 1 case in the geographic area, orange color: two cases in the geographic area, gray color: Ile-de-France area, with a zoom on the left of the map.

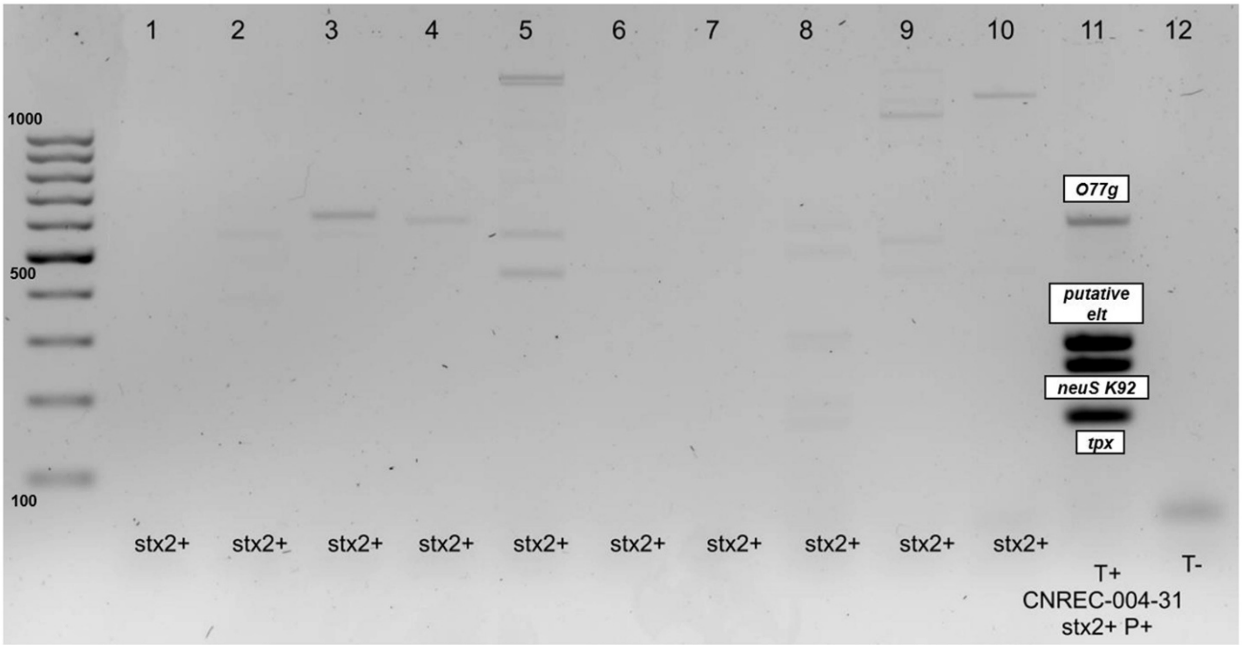

Appendix Figure 3. Quadruplex PCR targeting two chromosomal genes (*O77 g/*wzy and *neuS* K92) and two genes of the 134kb plasmid (putative *elt* and *tpx)*, performed on stool extracts from ten stx2+ patients unrelated to the outbreak (wells 1–10), with stool containing the outbreak isolate CNREC-004–31 as positive control (well 11) and water as negative control (well 12).

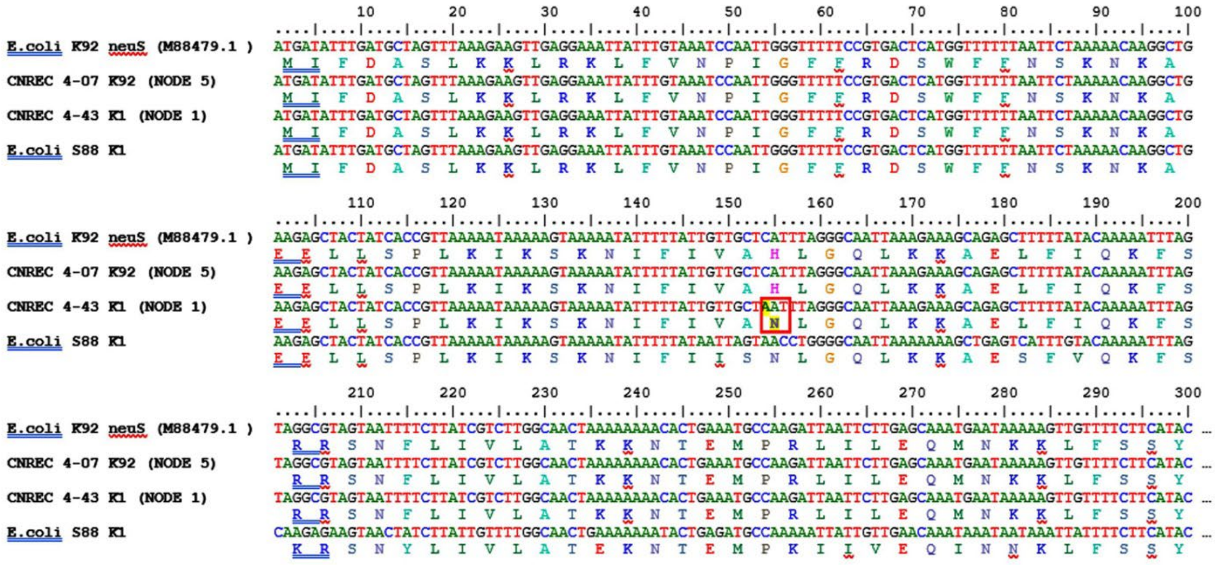

Appendix Figure 4. Alignment of the first 300 bases of the *neuS* gene showing the single nucleotide mutation C154A, resulting in an amino acid change (H52N) responsible for the capsular shift from K92 to K1 in strain CNREC 4–43 (highlighted in yellow and framed in red). *neuS* sequences of strains S88 (K1) and M88479.1 (K92) are shown as reference.

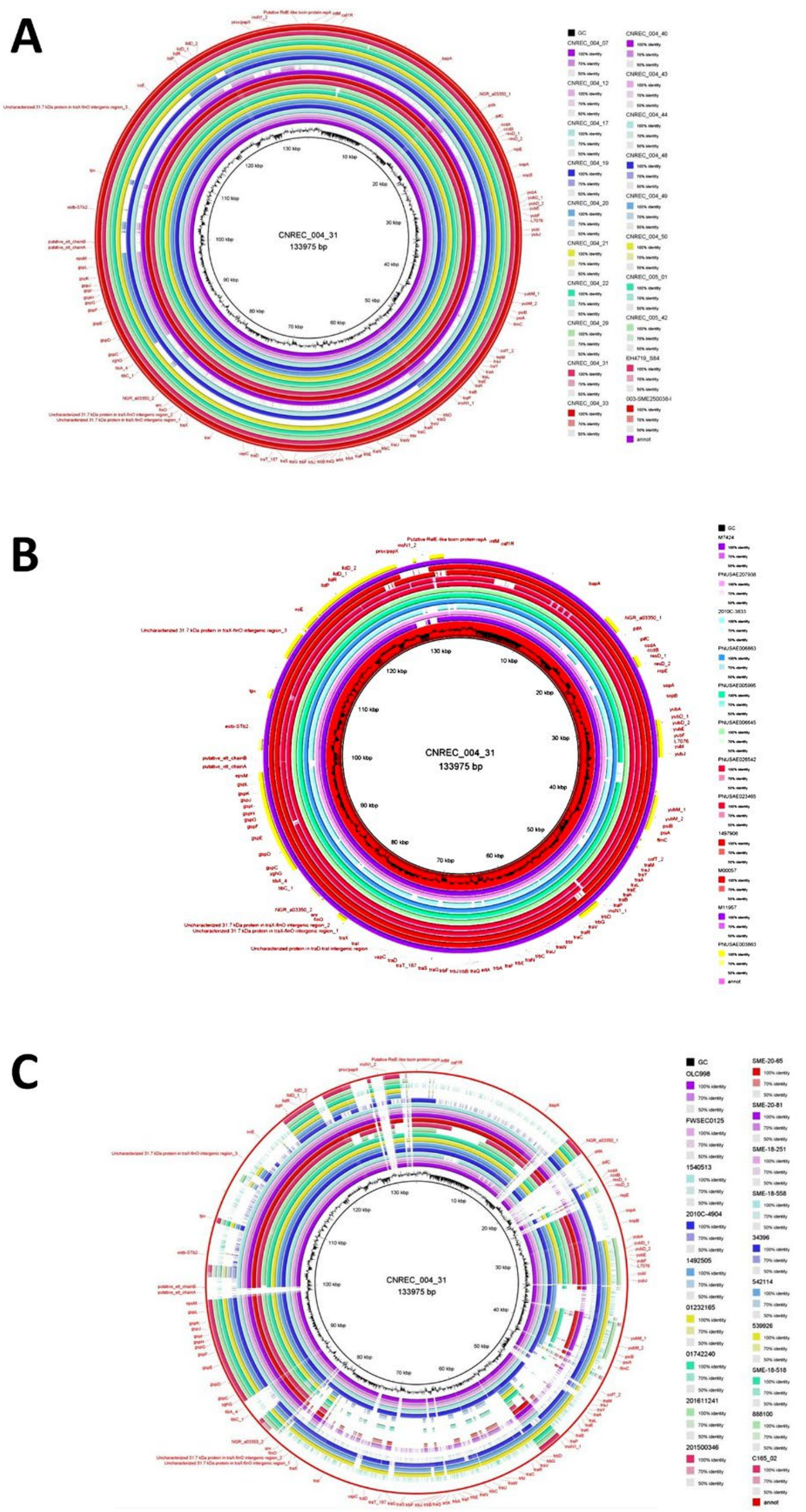

Appendix Figure 5. Alignment of 31 HC200 2073 isolate sequences against the 134 kb plasmid of isolate CNREC-004–31, performed using BRIG (https://github.com/happykhan/BRIG). A) Cluster A outbreak isolates (HC5 326896); B) Cluster A non-outbreak isolates; C) Cluster B isolates.

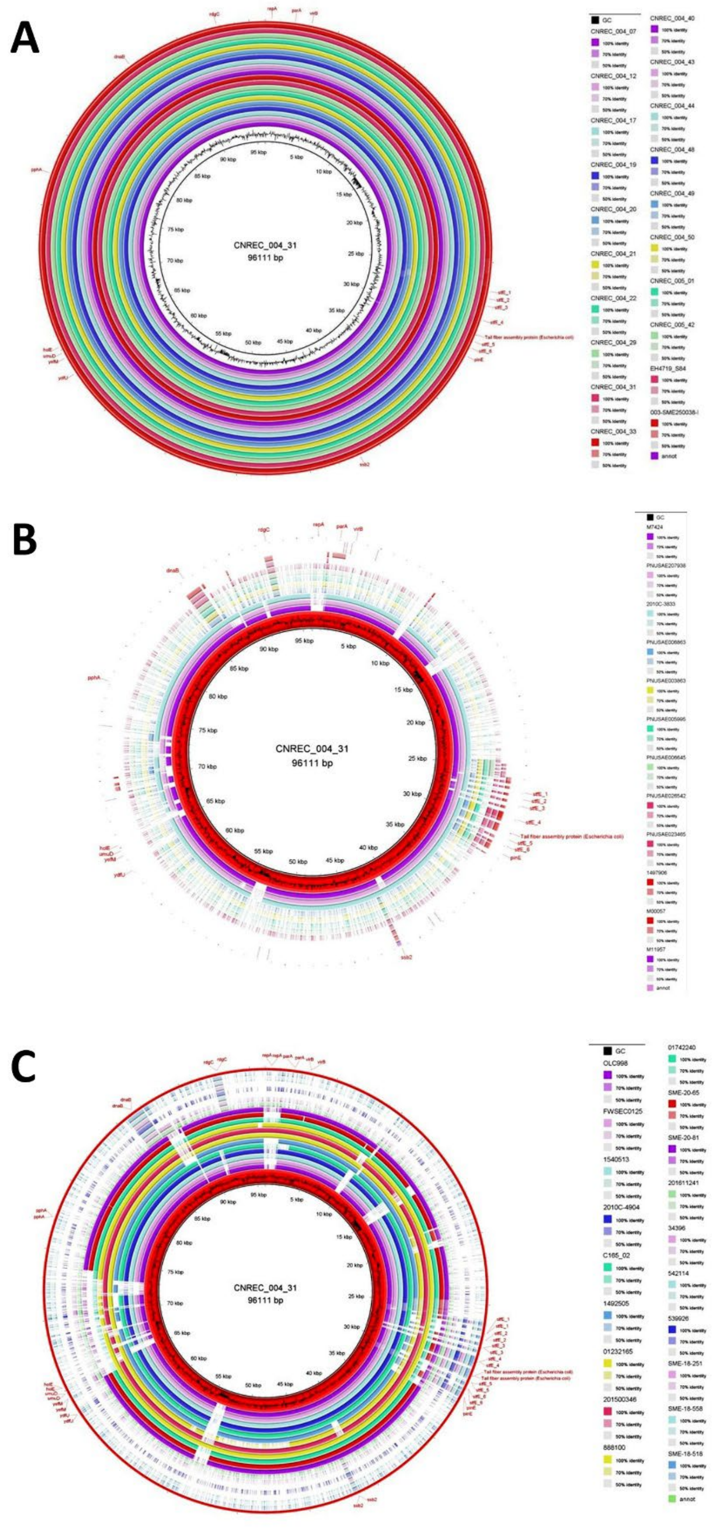

Appendix Figure 6. Alignment of 31 HC200 2073 isolate sequences against the 96 kb plasmid of isolate CNREC-004–31, performed using BRIG (https://github.com/happykhan/BRIG). A) Cluster A outbreak isolates (HC5 326896); B) Cluster A non-outbreak isolates; C) Cluster B isolates.

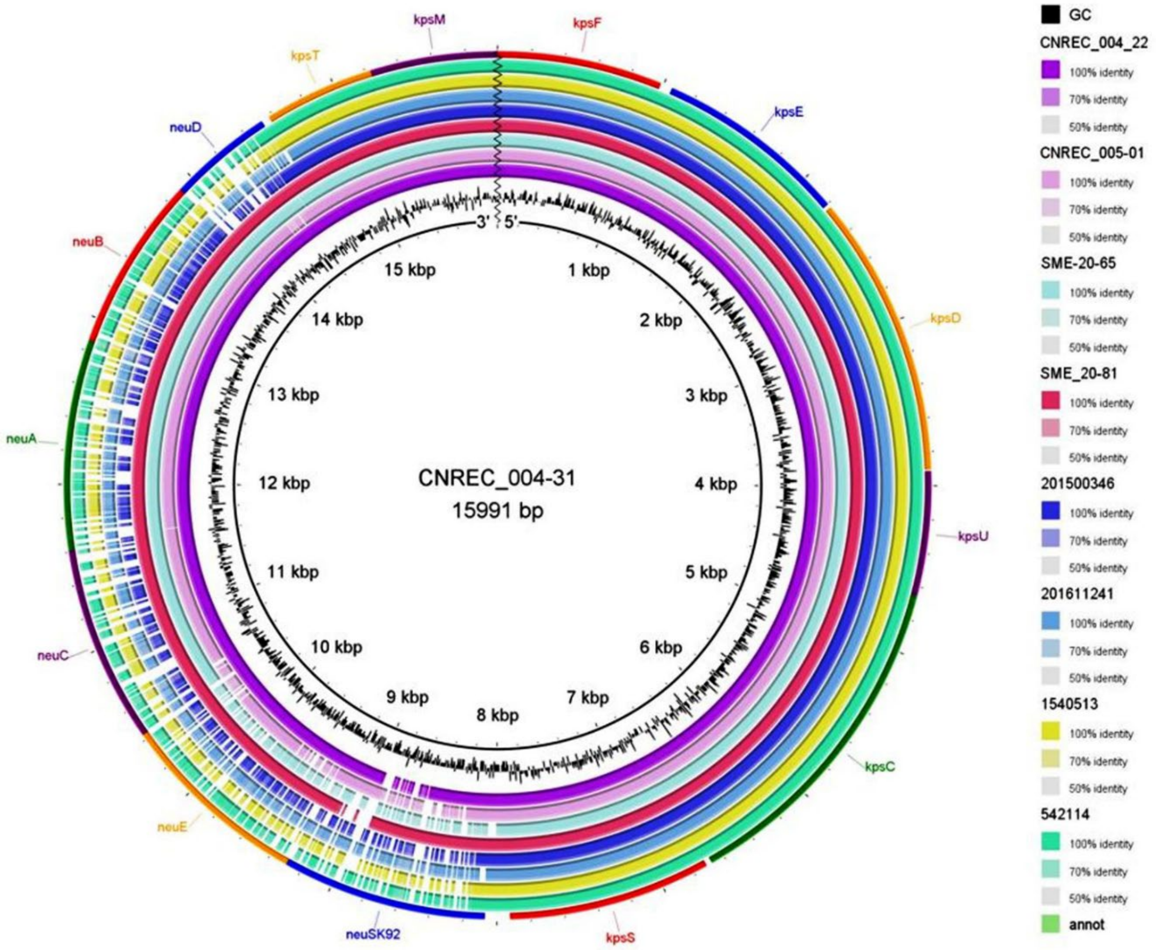

Appendix Figure 7. Alignment of K92 capsule operon of isolate CNREC_004–31 (15,991 bp) against several strains in which *neuC* and *neuS* genes are absent or ambiguous in Figure 1: CNREC_004–22, CNREC_005–01, SME-20–65, SME-20–81, 201500346, 201611241, 1540513 and 542114. The wavy line indicates the artificial junction between the 5′ and 3′ termini, as the sequence is linear but displayed as circularized for visualization purposes.
